# Family members’ perceptions of pain behaviors and pain management of adult patients unable to self-report in the intensive care unit: A qualitative descriptive study

**DOI:** 10.1080/24740527.2018.1544458

**Published:** 2018-11-26

**Authors:** Melissa Richard-Lalonde, Madalina Boitor, Sarah Mohand-Saïd, Céline Gélinas

**Affiliations:** a Ingram School of Nursing, McGill University, Montreal, Quebec, Canada; b Centre for Nursing Research and Lady Davis Institute, Jewish General Hospital, Montreal, Quebec, Canada; c Faculty of Medicine, McGill University, Montreal, Quebec, Canada

**Keywords:** assessment, family, intensive care, pain, proxy report, qualitative

## Abstract

**Background:**

Current guidelines suggest that family members be consulted in the pain assessment process of patients unable to self-report. However, little is known regarding family members’ perceptions of their loved one’s pain behaviors and pain management.

**Aims:**

This qualitative descriptive study aimed to describe family members’ perceptions of pain behaviors and pain management in critically ill hospitalized patients admitted to an intensive care unit and unable to self-report.

**Methods:**

A qualitative descriptive design was used. This study was conducted in a medical–surgical intensive care unit in Canada. Family members of nonverbal adult patients participated in a semistructured interview regarding their perceptions of pain behaviors and pain management in the intensive care unit.

**Results:**

Ten family members with a nonverbal loved one admitted to the intensive care unit participated. Family members agreed on the presence of pain in the intensive care unit and reported being proactive and applying nonpharmacological interventions to help palliate pain of their loved one. Although family members identified behavioral indicators such as grimace, limb movement, and verbal complaints to assess pain in their loved one, the majority were unsure of their ability to detect pain.

**Conclusions:**

Family members have intimate knowledge of their loved one and could be invited to share their perceptions of their loved one’s pain when they feel confident to do so.

## RÉSUMÉ

**Contexte**: Les lignes directrices actuelles suggèrent que les membres de la famille soient consultés lors du processus d’évaluation de la douleur des patients incapables de fournir une auto-évaluation. Toutefois, on sait peu de choses au sujet de perceptions des membres de la famille au sujet des comportements de leur proche relativement à la douleur et à la prise en charge de la douleur.

**But**: Cette étude descriptive qualitative avait pour but de décrire la perception des membres de la famille des comportements relatifs à la douleur et à la prise en charge de la douleur chez des patients en état critique de santé admis dans une unité de soins intensifs et incapables de fournir une auto-évaluation.

**Méthodes**: Un devis descriptif qualitatif a été utilisé. Cette étude a été menée dans une unité de soins intensifs médicale-chirurgicale au Canada. Les membres de la famille de patients adultes non verbaux ont participé à une entrevue semi-structurée concernant leur perception des comportements relatifs à la douleur et à la prise en charge de la douleur à l'unité des soins intensifs.

**Résultats**: Dix membres de famille ayant un proche non verbal admis à l’unité des soins intensifs ont participé. Les membres de famille étaient d’accord pour dire que la douleur était présente dans l’unité des soins intensifs. Ils ont aussi dit être proactifs et appliquer des interventions non pharmacologiques pour aider à soulager la douleur de leur proche. Bien que les membres de la famille ont identifié des indicateurs comportementaux comme une grimace, le mouvement d’un membre ou des plaintes verbales pour évaluer la douleur de leur proche, la majorité d’entre eux n’étaient pas certains de leur capacité à détecter la douleur.

**Conclusions**: Les membres de la famille ont une connaissance intime de leur proche et pourraient être invités à partager leur perception de la douleur ressentie par leur proche lorsqu’ils se sentent à l’aise de le faire.

## Introduction

Pain is a common occurrence among adult patients admitted to the intensive care unit (ICU). Pain is experienced during routine ICU procedures such as chest tube and wound drain removal and arterial line insertion^1^ but also when patients are at rest.^2,3^ Several barriers pose challenges to ensuring adequate pain management for all ICU patients, including the failure to assess and acknowledge the presence of pain, especially if patients are unable to self-report.^4,5^ The American Society of Pain Management Nursing and the Registered Nurses’ Association of Ontario recommend that family members be consulted in the pain assessment of patients unable to self-report because of their unique point of view and their familiarity with the patient, which could enable them to identify subtle pain responses easily missed by clinicians.^6,7^

In a large study of 2645 patients and family members, family surrogates could correctly estimate the presence/absence of pain 73.5% of the time (kappa = 0.47, 95% confidence interval, 0.44–0.50), overestimated it 16.8% of the time, and underestimated it 9.7% of the time, with a higher sensitivity (88.6%) observed with severe pain intensity.^8^ One study comparing the self-reports of pain of 245 ICU patients to the proxy reports of family members, nurses, and physicians showed that family members’ ratings of pain intensity were more consistent with patient ratings than either nurses’ or physicians’ ratings, thereby highlighting the valuable role that family members can have in the pain assessment process.^9^ However, both studies included only patients able to self-report. These studies indicate acceptable correlations between reports of pain from family members and patients who are capable of verbal self-report. They also show the value of recommendations to consider involving family members in pain assessment in the ICU. However, little is known regarding family members’ perceptions of (1) pain in a nonverbal critically ill loved one, (2) effective pain relief strategies, and (3) their confidence in their ability to detect pain in their nonverbal loved one.

To our knowledge, only one study (*n* = 7) examined family members’ perceptions of their nonverbal loved one’s pain behaviors while hospitalized in the ICU after a traumatic brain injury.^10^ In this study, family members participated in a semistructured interview to describe their perceptions of their nonverbal loved one’s pain responses and rated the relevance of a list of pain behaviors using a self-administered questionnaire. Family members rated facial expressions (i.e., tearing, brow lowering) and body movements (i.e., attempting to reach or touching pain site, restlessness) as the most relevant behaviors for the detection of pain and described other facial expressions (e.g., lip movements) and reactions (e.g., not moving) related to their intimate knowledge of their loved one. Such findings support the family’s unique contribution in the assessment of pain in the critically ill patient unable to self-report and their potential to assist clinicians in this challenging endeavor. Although the study^10^ presents findings regarding family members’ perceptions of pain behaviors in nonverbal ICU patients, previous studies show that patients with traumatic brain injuries express different behaviors than their medical–surgical counterparts.^11,12^ Given the differences between patient populations, studies are needed to assess family members perceptions of pain behaviors in nonverbal patients admitted to a medical–surgical ICU.

## Methods

### Research objectives

The present study aimed to describe family members’ perceptions of the presence of pain, pain behaviors, and pain management in ICU patients unable to self-report.

### Research design

A qualitative descriptive design was used to gain a better understanding of family members’ views because, to date, this topic has been largely understudied. A qualitative descriptive design was selected to stay close to the data and use low levels of interpretation.^13^

### Setting and sample

This study took place in the medical–surgical ICU of a university-affiliated health care center in Canada with an open visiting policy except during changes of shift when nursing reports are given. Access to the ICU is granted upon request from the unit agent and responsible nurse. Family members are generally allowed to be present during care procedures after consultation with the ICU team and as per their preference. Family members were defined as someone living with the patient at the time of hospital admission, an individual who the patient saw regularly, or one who played a significant role in the patient’s life for a minimum of one year, which is consistent with the definition used by Lefebvre et al.^14^ Eligible family members were aged 18 years or more, English or French speaking, had a loved one in the ICU, and were present at the patient’s bedside at least three times during the current ICU stay. Furthermore, to be eligible, their loved one had to be admitted to the ICU for a minimum of three days and unable to self-report pain verbally or through gestures (e.g., head nodding, hand squeezing, blinking). Family members were excluded if their loved one was investigated for brain death, suffered from quadriplegia, or received neuromuscular blocking agents, because these interfere with the expression of behaviors. Using convenience sampling, a total of ten family members was sought because this is considered appropriate for reaching data saturation in qualitative interviews.^15^

### Ethics approval

The hospital Research Ethics Board approved the conduct of this study. With the help of the nursing team, the research personnel first assessed the patient’s eligibility criteria and, if considered eligible, screened family members for inclusion . Eligible family members were approached by the nurses, who verified their interest in participating in the study. If willing to participate, family members were met in person by the research personnel, who introduced the study and obtained their written consent. Family members received no incentives or compensation for their participation.

### Data collection

Consenting family members participated in a short individual in-person interview using a semistructured interview guide inspired by the PQRSTU (Provoking/Palliating, Quality, Region, Severity, Understanding) mnemonic,^16^ which is commonly used to describe and assess pain in patients able to self-report. The same interview guide was used for all family members, and prompting questions were adapted during each interview to encourage the elaboration of ideas.

Questions were directed to family members to explore their unique perception of pain in their nonverbal loved one. The interview guide used open-ended questions^1010^ and aimed to solicit family members’ input regarding the context surrounding the presence of pain (i.e., Provoking factors) and effective strategies for pain relief in the ICU (i.e., Palliating factors). Moreover, they were asked to describe the behaviors they observed to be related to pain (i.e., Quality, Region) including their specificity for pain (i.e., Specificity) and timing of occurrence (i.e., Timing). Finally, they were asked to elaborate on their confidence in their ability to detect pain (i.e., Understanding) in their loved one when unable to self-report. The interview guide was translated into French by consensus between the medical student (SMS) and the principal investigator (CG) who is a French-native speaker. Individual interviews were held for 5 to 20 min with an average interview time of 10 min, took place in a private room at the study site, and were audio-recorded for verbatim transcription.

The interviews were conducted by two female research team members: a medical student with undergraduate research training (SMS) and a nurse pursuing doctoral studies (MRL). The interviewers had no experience working in the ICU or on research projects related to family participation in symptom assessment. Prior to data collection, interviewers had a limited understanding of families’ perceptions of pain in their critically ill loved one given that this topic has remained largely understudied in the adult ICU. There were no prior relationships between participating family members and interviewers who met for the first time in the context of this research study.

### Data analysis

The QDA Miner (Provalis Research) software was used to manage and organize interview transcripts. Content analysis was used to analyze the interview transcripts.^17^ Using an inductive approach, one interviewer (MRL) and a nurse with doctoral training (MB) generated codes from specific sections of the transcripts and then merged them to form themes independently. To ensure that findings were grounded in evidence, each code was supported with quotes. Their findings were discussed with the principal investigator (CG), a nurse with doctoral training and prior experience in conducting and analysing qualitative research, until consensus was reached to ensure a comprehensive and shared understanding of the messages conveyed by family members. The principal investigator (CG), the interviewer (MRL), and the nurse with doctoral training (MB) had previous knowledge of common behaviors used in behavioral pain scales for nonverbal ICU patients. This knowledge was used to structure data analysis regarding pain behaviors into five main domains: facial expression, body movement, vocalization/ventilator compliance, muscle tension, and autonomic responses. The PQRSTU mnemonic was used as a guide to present the qualitative findings and provide a priori overarching coding categories.

## Results

### Sample

A total of 17 eligible family members were approached for participation in the study, of whom five refused to participate because of feeling overwhelmed and tired (*n* = 3), not knowing the patient well enough to participate in the study (*n* = 1), and not being available (*n* = 1). Twelve family members consented to participate, but two did not show up for the interview. The sample included ten family members of patients admitted in the ICU between July and September 2016 and January and May 2017 (Table 1). Data collection was stopped after reaching ten family members given that, in the context of this descriptive study, a general understanding of families’ perceptions of pain in their nonverbal loved one was achieved. Furthermore, they were a balanced mix of males and females and representative of the family members who regularly visit ICU patients.

**Table 1.** Demographics and descriptive statistics of participating family members.

Demographics	Frequency	(%)		
Gender				
Male	4	40.0		
Female	6	60.0		
Education level (completed)				
Primary	0	0.0		
Secondary	2	20.0		
Collegial (CEGEP)	2	20.0		
University	6	60.0		
Relationship				
Spouse	4	40.0		
Child	6	60.0		
Living with patient prior to ICU admission				
No	5	50.0		
Yes	5	50.0		
Descriptive statistics	Mean (SD)	Median	Minimum	Maximum
Age	59.60 (20.21)	49.50	36	89
Length of relationship between patient and family member (years)	49.10 (8.17)	49.50	36	65
Number of visits at bedside	8.50 (5.78)	6.00	3	20
Average length of visits (min)	225.00 (190.53)	165.00	45	600

CEGEP = Collège Enseignement Général Et Professionnel; ICU = intensive care unit.

Family members who participated in the present study and reported on ICU patients were either their spouse (40%) or child (60%), with a median age of 49.5 years, and with whom they had a relationship for a median of 49.5 years. Half of family members were living with the patient prior to the ICU admission. They visited the patient on average 2.75 h per day at the time of the interview. Patients (*n* = 10) were aged between 71 and 87 years old, with a median age of 76.50 years. Four family members participated in the interview in French, and the verbatim interviews presented in this article were translated in English. Four patients were mechanically ventilated, but all were unable to self-report during data collection.

Three main themes and several subthemes emerged from the interviews conducted with family members and are presented below.

### Family members’ perceptions of pain in their loved one

#### Provoking factors

All family members could recall at least one event in the ICU during which they believed that their loved one experienced pain. The most common reported causes of pain were the presence of a tube (e.g., endotracheal, urinary; *n* = 5), followed by endotracheal suctioning (*n* = 4), repositioning (*n* = 3), coughing (*n* = 3), turning (*n* = 2), and the use of restraints (*n* = 2). Other events that were identified once included injection, dressing change, surgery, cancer, back tapping, seizure, and touching. For example, family members said, “When it came to needles and invasive types of procedures, yes, I believe there was pain” (family member #3) and “When they were suctioning tubing around … she looked much with … excruciated with pain” (family member #4).

Whereas most of the causes of pain relate to commonly performed procedures in the ICU, four family members (40%) also reported believing that their loved one experienced pain at rest.

#### Palliating factors

Almost all family members (*n* = 9, 90%) mentioned medication such as analgesics (e.g., morphine, acetaminophen) and sedatives (e.g., propofol) to relieve pain. Three family members associated the use of sedation with actual pain relief: “[Does she feel pain] When she is at rest? Well, no, with the propofol, if she feels pain, well then they increase [the dose]” (French verbatim translated into English, family member #1). “But I know that he was sedated, so he wasn’t in any pain.” (family member #6)

The most common palliating nonpharmacological approaches reported were family visits (*n* = 4, 40%) and physical touch such as hand-holding and massage of the hands and feet (*n* = 4, 40%). Communication in the form of gentle talking (*n* = 2) and giving encouragements (*n* = 2) has also been raised as a comforting strategy employed by family members. Other reported palliating factors included repositioning, reading (to the patient), using a humid cloth, putting a heater in the room, loosening of restraints, and use of the oral swab to moisten the patient’s mouth. One family member elaborated on multiple nonpharmacologic methods for palliating her father’s pain: “loving touch … hand-holding … head and my arm around my father’s shoulders and a lot of physical touch—hand massage, foot massage, leg rubbing. And talking about positive memories—expressing love and gratitude—[…] also to encourage my father and say that he is doing well—he is getting better and without denying the situation” (family member #3).

### Pain behaviors observed by family members

#### Facial expression

Most family members (*n* = 8, 80%) identified behaviors related to facial expression as being indicative of their loved one’s pain. The most commonly reported facial expression pain behavior was grimacing (*n* = 4, 40%) followed by closing eyes (*n* = 2, 20%): “Well when they are suctioning her, she does have some resistance … she will lift up her head a bit; she’ll grimace …” (family member #4).

Other facial expressions reported once included eyes being tightly closed, eye opening, brow lowering, and movement of the lips.

#### Body movements

Overall, all family members referred to some sort of body movement as indicative of their loved one’s pain. Family members (*n* = 5, 50%) noticed that patients would attempt to remove equipment such as endotracheal and tracheostomy tubes when in pain: “He had a trach on him and he kept on pulling that. So, we had to make sure that we were there for him, and that was very painful” (family member #5).

Other reported body movements were related to hands and arms, with descriptions ranging from flailing arms to grasping hands: “She will take … her right hand—she moves a lot and push away. When … she was a little sedated people were trying to touch her or reposition her—she pulls her right arm out … and flails it out—she tries to put some space between her and the other person—she tries … contortion herself” (family member #4).

Some family members also noticed full body movements such as “contortion of the body,” “bending over,” and “lifting the body up.” One family member described: “She was almost writhing in bed because she was in pain” (French verbatim translated into English, family member #8)

#### Ventilator compliance

At the time of the interview, only four patients (40%) were mechanically ventilated. One family member referred to the ringing ventilator alarm as indicative of pain: “That’s for coughing. … She looks like she’s in pain now and when she coughs” (family member #4).

#### Vocalization

Verbal complaints were the most commonly noticed responses to pain (*n* = 5, 50%), followed by moaning (*n* = 2, 20%) and general sounds such as “groaning” and “grunting”: “He would literally tell me: ‘Stop them touching me … tell them to stop touching me. Because it bothers me. … I’m in pain’” (family member #5 recalling when the patient was able to speak) and “Verbally … my mother does not speak at all. When she is in pain, she verbally goes, “Ouh! Ouh!” And you can tell she is in a lot of pain” (family member #4).

#### Muscle tension

Three family members (30%) had thought of muscle tension as being indicative of their loved one’s pain. For example, family member #3 expressed it as “tensing” and observing “movements of resistance.”

#### Autonomic responses

Three family members identified pain indicators corresponding to autonomic responses such as tearing (*n* = 1), increased blood pressure (*n* = 1), and being cold (*n* = 1).

### Family members’ perceptions of pain assessment

#### Family members’ perceptions of their confidence in their ability to detect the presence of pain in their loved one

Family members provided varying responses with regards to their confidence in their ability to detect pain in their loved one. In general, family members reported feeling either confident (*n* = 3, 30%) or not (*n* = 3, 30%) in their ability to detect pain in their loved one or vacillated between the two stances throughout the interview (*n* = 3, 30%). One family member did not find the question relevant because she stated that it was unequivocal that anyone postsurgery must be experiencing pain: “As she is coming out of surgery … we assume when you get out that yes [there is pain because] there is inflammation” (French verbatim translated into English, family member #8).

Family members who reported being confident in their ability to detect the presence of pain in their loved one referred to their close connection with the patient to be instrumental in assessing pain:

Yes micro … family members are spending the time with a loved one and also you know your family member … you know your family and you know what the usual expression is—and also there is a bond of love and a desire to be able to communicate. So you look for any indication because if the person is having trouble communicating in normal ways—you tune in to any type of sign; any micro expression and any movement. Because you’re looking for … and you want that connection, so that’s information that maybe a nurse doesn’t have … doesn’t know the patient and doesn’t have time … but you think that’s important. (family member #3)

Another family member described that knowing the person well facilitates distinguishing painful from non-painful behaviors:

Right away because I spend every day with her and I know when she’s at rest or when she’s in pain even before when she was at the home—I see her every day—and then if she had a little trouble breathing or she looked there was a pain expression on her face—just little things … she looks at me or facial expressions she’ll make with her eyes—she would close her eyes and she would have a little pain shooting. I would tell the nurse that she is in pain. She would say: “How do you know?”—I know she wasn’t like that yesterday and she acts a little bit as if she is in pain. It is nonverbal things … but I can notice and say: Hey! Because I’m there every day and I see her behavior and I know when she’s at rest and when she’s happy and when she’s in pain. (family member #4)

Three family members reported lacking confidence in their ability to discern whether their loved one was in pain. One family member stated that she did not know how her father could express pain when he was unable to communicate: “If he did not talk—I don’t know how I … how we would know. Probably he would do gestures. I don’t know” (French verbatim translated into English, family member #9).

Another family member, when referring to his wife’s secretions being suctioned, said: “Now I don’t know whether it is painful or disturbing … just disturbing. I’ve never tried it myself so I don’t know but I suspect it is disturbing to her … the patient” (family member #10).

Three family members vacillated between feeling and not feeling confident in knowing whether the patient was in pain depending on the event they recalled and reported during the interview and defined the competency of pain assessment as context specific.

#### Family members’ perception of clinicians’ abilities to detect pain in their loved one

Family members reported comparing their own evaluations of their loved one’s pain with that of physicians and nurses. In some instances, this comparison resulted in the family members questioning their own observations and judgments with regards to the presence of pain: “I am under the impression that I was mistaken in my judgment—according to the doctor—because he says that she is not suffering” (French verbatim translated into English, family member #7). Another family member stated: “She still does some sort of a grimace. Is it convulsive? We don’t know. I am under the impression … I always ask [about pain] and they tell me: “No.” So, it reassures me. But … is it because they want to reassure me? That, I don’t know. In the end, it’s quite possible, isn’t it?” (French verbatim translated into English, family member #1).

Whereas some family members felt that clinicians influenced their own pain assessments, three family members expressed doubt regarding the clinicians’ abilities to detect pain: “One is allowed extra pain medication when she is in pain and so that will enable the nurse to give her extra pain medication, right, when my mother is in pain. I know she [the nurse] would not know to give my mother [pain medication]” (family member #4).

#### The importance of pain assessment and management from the perspective of family members

Family members acknowledged the importance of pain assessment and relief; however, in certain circumstances, proceeding with medical procedures and protecting the patient’s safety become the priority:

I guess watching him … seeing the pain through his eyes and then expressing myself I mean it didn’t happen here but somewhere else, you know, I would say it’s hurting him and nothing has to be done so you have to back yourself up to say: OK! Is it the pain or it has to be done? So you go by the logic: OK it has to be done! So you’re looking at the pain and it’s hurtful to see that. (family member #5)

I think it might be useful but I’m saying ultimately the patient safety is paramount and it is what I really care about. But anything that can be done to alleviate the … to make the patient comfortable and to be conducive to their healing and their recovery, and obviously, again, patient’s safety is paramount … and more important than comfort. If they pricked somebody finger too hard, you know, they’ll still live. (family member #2)

## Discussion

To our knowledge, this is the first study to explore family members’ perceptions of pain behaviors and pain management in the medical–surgical ICU. Family members have already been solicited as proxy reporters of their loved one’s pain^9^; however, little is known about their perceptions of pain in nonverbal ICU patients. The results of the present study suggest that family members concur on the presence of pain in their nonverbal loved one admitted in the ICU, rely on behavioral indicators to assess pain, and are proactive in attempting to relieve pain using nonpharmacological intervention, yet most lack confidence in their ability to detect the presence of pain.

All family members perceived at least one event when they believed that their loved one was in pain, and this occurred not only during a procedure but also when patients were at rest. Their observations are consistent with reports of procedural pain in the ICU^1^ and that approximately half of ICU patients experience pain at rest (*n* = 117/230, 51%).^2^ Spontaneously, family members looked for specific behaviors related to their loved one’s facial expression, body movements, muscle tension, and vocalization to detect pain. These results are contrary to what has been observed in family members who were asked to evaluate pain in loved ones with dementia.^18^ Although many persons with dementia have limited ability to communicate their pain verbally, as is the case with many adult ICU patients, family members of persons with dementia disregarded or did not notice nonverbal pain cues. This difference in study findings could be explained by the differing contexts (long-term care facilities vs. ICU) and the expectation for the presence of pain in the ICU.

The most frequently reported behaviors (i.e., grimacing, contortion of the upper face, limb movement, agitation) are also included in behavioral scales developed for pain assessment in the critically ill unable to self-report, such as the Critical-Care Pain Observation Tool (CPOT),^19^ and are worth half of the points on the total CPOT score. The facial expression features reported by family members are also consistent with those observed by trained experts who used the Facial Action Coding System in a surgical–trauma ICU and a medical ICU.^20^ Only some family members relied on the alarms of the mechanical ventilator as indicators of pain, possibly due to the lack of familiarity with the origin and meaning of the various ICU alarms. Similar behaviors were identified by family members of nonverbal ICU patients with traumatic brain injury to be relevant for pain assessment in this vulnerable population.^10^ Such observations come in support of the current clinical practice recommendations to use behavioral pain scales with critically ill patients who are unable to self-report.^6,21^

Family members identified pharmacological analgesia to palliate pain and, interestingly, almost one third of them associated the use of sedation with pain relief because they noticed nurses increasing the infusion of propofol when their loved one appeared to be in pain. Family members reported employing various nonpharmacological interventions to relieve pain, ranging from physical touch (e.g., massage) to family presence and emotional support. These family-driven interventions indicate that family members strive to participate in pain management and rely on their intimate knowledge of the patient to select the interventions that are more likely to palliate pain. The use of nonpharmacological interventions for pain management in the ICU has received favorable input from family members, patients, and ICU nurses in terms of their usefulness, relevance, and feasibility in the ICU given their ease of administration and safety.^22^ Nurses and family members have also been shown to respond to procedural pain (i.e., during turning) by using nonpharmacological interventions such as calming voice, touch, massage, and family presence.^23^

Only a small proportion of family members felt confident in their ability to detect pain in their nonverbal loved one due to their close connection and intimate knowledge of the patient. The unique history of each family member with the patient prior to the ICU admission could explain the mixed reports regarding their confidence with pain detection, but larger studies are needed to explore the influential factors of family members’ pain assessment self-efficacy in the ICU. Whereas clinical practice guidelines^6,7^ and previous research^9^ suggest consulting family members for pain assessment in the nonverbal critically ill patient, the present results indicate that clinicians should be cognizant of the fact that pain assessment can be challenging even for family members. Providing family members with guidance on the use of behavioral scales for pain assessment could help overcome these challenges, as observed with informal caregivers of seniors with dementia.^24^

Some family members expressed doubt regarding the accuracy of pain assessments performed by ICU clinicians. Based on previous studies, family members share a different perception of pain than ICU clinicians because they tend to score pain intensity higher^9^ and pain control lower than nurses and physicians.^24^ Such differences in pain perception could explain family members’ questioning of clinicians’ assessments and underscore the need for effective communication between clinicians and families to ensure that pain is detected in the nonverbal critically ill.

Throughout the interview, family members appeared to attribute a great importance to adequate pain relief because they were attuned to subtle behavioral indicators of pain, advocated for better pain relief, and tried nonpharmacological interventions; however, they also acknowledged the need to proceed with ICU procedures that can provoke pain. Witnessing their loved one in pain triggered distress in family members because they felt they had to choose between patient safety and pain relief. Family members of ICU patients have been shown to experience high levels of emotional distress related to unmet emotional or information needs^25,26^ and to be at risk for the development of posttraumatic stress disorder and depression.^27–29^ One of the most important needs identified by family members is being reassured that the best care is provided for the patient,^30^ which calls forth the need to inform them of the aims and steps of the procedures performed to their loved one as well as any measures taken to minimize procedural pain.

### Limitations

One limitation of this descriptive study is that participating family members were either the child or spouse of the patient from a single center in Canada, thereby leaving the perspectives of other family members such as siblings or cousins unknown. A larger sample size would have enhanced the generalizability of results by including family members from multiple ICUs and of more diverse cultural backgrounds. Future research with larger and more heterogeneous samples is needed to complement this study and help formulate guidelines regarding the context of family involvement in pain assessment in the ICU. Furthermore, family members participated on a voluntary basis, which raises the possibility of self-selection bias.

## Conclusion

This is one of the first studies to explore family members’ perceptions of pain behaviors and pain management of their nonverbal loved one admitted in the ICU. Family members concur on the presence of pain during ICU procedures but also at rest and try nonpharmacological interventions to palliate pain. They rely on behaviors in their attempts to assess pain, yet most are unsure of their ability to assess pain. Family members could participate in the pain assessment of their loved one when they feel confident to do so.^21^ Future research is needed to explore the views of family members of more diverse cultural backgrounds.
